# Bright Transparent Scintillators with High Fraction BaCl_2_: Eu^2+^ Nanocrystals Precipitation: An Ionic‐Covalent Hybrid Network Strategy toward Superior X‐Ray Imaging Glass‐Ceramics

**DOI:** 10.1002/advs.202304889

**Published:** 2023-10-18

**Authors:** Qunhuo Liu, Peng Ran, Weilin Chen, Nian Shi, Wei Zhang, Xvsheng Qiao, Tingming Jiang, Yang (Michael) Yang, Jinjun Ren, Zhiyu Wang, Guodong Qian, Xianping Fan

**Affiliations:** ^1^ State Key Laboratory of Silicon Materials School of Materials Science and Engineering Zhejiang University Hangzhou Zhejiang 310027 China; ^2^ State Key Laboratory of Modern Optical Instrumentation College of Optical Science and Engineering Zhejiang University Hangzhou Zhejiang 310027 China; ^3^ Key Laboratory of Materials for High Power Laser Shanghai Institute of Optics and Fine Mechanics Chinese Academy of Sciences Shanghai 201800 China; ^4^ School of Energy and Power Engineering Chongqing University Chongqing 400044 China

**Keywords:** BaCl_2_:Eu nanocrystals, ionic‐covalent hybrid network, scintillator, transparent glass ceramics, X‐ray imaging

## Abstract

Metal halide crystals are bright but hygroscopic scintillator materials that are widely used in X‐ray imaging and detectors. Precipitating them in situ in glass to form glass ceramics (GCs) scintillator offers an efficient avenue for large‐scale preparation, high spatial resolution, and excellent stability. However, precipitating a high fraction of metal halide nanocrystals in glass to maintain high light yield remains a challenge. Herein, an ionic‐covalent hybrid network strategy for constructing GCs scintillator with high crystallinity (up to ≈37%) of BaCl_2_: Eu^2+^ nanocrystals is presented. Experimental data and simulations of glass structure reveal that the Ba^2+^‐Cl^−^ clustering promotes the high crystallization of BaCl_2_ nanocrystals. The ultralow phonon energy (≈200 cm^−1^) of BaCl_2_ nanocrystals and good Eu reduction effect enable high photoluminescence inter quantum efficiency (≈80.41%) in GC. GCs with varied crystallinity of BaCl_2_: Eu^2+^ nanocrystals demonstrate efficient radioluminescence and tunable scintillator performance. They either outperform Bi_4_Ge_3_O_14_ single crystal by over 132% steady‐state light yield or provide impressive X‐ray imaging resolutions of 20 lp mm^−1^. These findings provide a new design strategy for developing bright transparent GCs scintillators with a high fraction of metal halide nanocrystals for X‐ray high‐resolution imaging applications.

## Graphical abstract

An ionic‐covalent hybrid glass network is designed to fabricate transparent and stable glass ceramic scintillator with high crystallinity of BaCl_2_: Eu nanocrystals, which exhibits strong radioluminescence characterized by a light yield 132% of Bi_4_Ge_3_O_14_ single crystal. Besides, the transparent glass ceramic scintillator can be applied as X‐ray imaging application with a high spatial resolution of 20 lp mm^−1^.

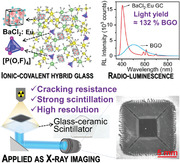

## 1 Introduction

Scintillators are materials that convert ionizing radiation into visible light, which have found wide applications in medical radiography,^[^ ^1^ ^]^ non‐destructive inspection,^[^ ^2^ ^]^ nuclear physics,^[^ ^3^ ^]^ cosmic radiation detection,^[^ ^4^ ^]^ etc. Recent decades have witnessed the application of typical inorganic halide scintillators, including alkali‐metal halides, alkali‐earth halides, rare‐earth halides, metal halide perovskites, and lanthanide‐doped halides. The discovery of NaI:Tl^+^ and CsI:Tl^+^ has opened up many X‐ray scintillators’ applications,^[^ ^5^ ^]^ and later LaBr_3_: Ce^3+^ that has better temperature stability, faster decay and higher energy resolution was successfully commercialized.^[^ ^6^ ^]^ At present, the highest scintillation light yield of up to 120 000 ph Mev^−1^ was achieved in SrI_2_: Eu^2+^.^[^ ^7^ ^]^ Recently, CsPbX_3_ and lead‐free halide perovskites have attracted enormous attention for ultrasensitive X‐ray detectors and real‐time X‐ray imaging because of their high absorption cross section for X‐ray and ultra‐fast scintillation.^[^ ^8^ ^]^ Among these scintillators, Eu^2+^ activated alkali‐earth halides (CaI_2_,^[^ ^9^ ^]^ SrI_2_,^[^ ^10^ ^]^ BaCl_2_,^[^ ^11^ ^]^ BaBr_2_,^[^ ^12^ ^]^ BaI_2_,^[^ ^13^ ^]^ etc.) have been found to exhibit good stopping power and high light output. This was beneficial from two aspects: the intense luminescence originating from 5d‐4f spin allowed the transition of Eu^2+^ ions, and the available growth of high‐quality and un‐cracked alkali‐earth halide single crystals due to their small difference of thermal expansion coefficient in different crystal direction.^[^ ^14^ ^]^ However, most of the non‐fluorinated halide scintillation crystals suffer from sensitivity to both moisture (hygroscopic) and oxygen from the atmosphere, which can strongly deteriorate the scintillation performance.^[^ ^15^ ^]^ Simultaneously, it can cause a high cost for the use of high‐pure anhydrous raw materials, strict crystal fabrication conditions and hermetic packaging.^[^ ^15b^ ^]^

To solve the hygroscopic problems, transparent oxide glasses were employed as the scaffold for growing halide nanocrystal scintillator, which has proved to be an effective approach to improve the stability and spatial resolution for scintillator application.^[^ ^16^ ^]^ But this is achieved at a large cost of light yield because of the low fraction of halide nanocrystal scintillators in the oxide glass host. Indeed, the volatilization of halide in common oxide glass with high melting temperature and the limited halide solubility in oxide glass^[^ ^17^ ^]^ significantly inhibit the high crystallinity of halide nanocrystals in glass. Compared with the immiscible oxyhalide glass network, the fluoride glass network has a lower melting temperature and better compatibility with halide so that can accommodate more halide, and thus enable a higher fraction of halide nanocrystal scintillators. ZrF_4_‐based glass had been used to precipitate Eu^2+^‐doped BaCl_2_ nanocrystals and shows scintillator application.^[^ ^18^ ^]^ Whereas, the poor chemical stability, the economically unfavorable preparation method and the readily concomitant inactive crystalline phase in the ZrF_4_‐based glass hinder its further industrial application. AlF_3_‐based glass shows a higher chemical durability three orders of magnitude greater than ZrF_4_‐based glass.^[^ ^19^ ^]^ However, the poor glass forming ability has been a drawback to utilizing this glass for precipitating halide nanocrystals.

Here, we present a general strategy for the construction of chemically‐stable AlF_3_‐based glass ceramic (GC) scintillator with high metal‐halide crystallinity, which has an elaborately designed ionic‐covalent hybrid structure that is demonstrated by integrating experimental data and ab initio molecular dynamics (AIMD) calculations. Due to the high crystallinity of BaCl_2_ nanocrystals with ultralow phonon energy, the designed BaCl_2_: Eu GC exhibits a high internal quantum efficiency and a high scintillator light yield. Finally, the Eu^2+^ activated BaCl_2_ GC has been applied in high‐resolution X‐ray imaging for several objects.

## 2 Results and Discussion

**Scheme 1:**
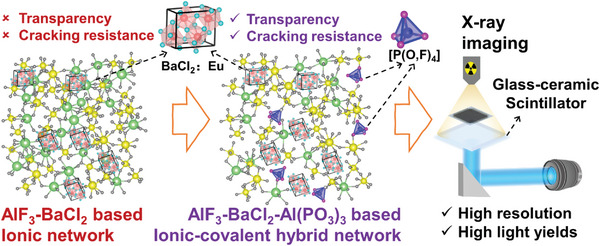
Illustration of the design and advantages of transparent AlF_3_‐BaCl_2_‐Al(PO_3_)_3_ based ionic‐covalent hybrid network containing with high fraction of BaCl_2_ nanocrystal scintillators for X‐ray Imaging application.

To precipitate high fraction of BaCl_2_: Eu nano‐crystalline scintillators in AlF_3_‐based glass scaffold, the material design strategy is set to construct an ionic‐covalent hybrid network structure (**Scheme** **1**) with: i) diverse mixed ionic polyhedra blended few covalent network‐forming polyhedra as the network skeleton; ii) homogenously nanoscale clustering of Ba^2+^ and Cl^−^ as the structural foundation of nano‐crystallization. According to the confusion rule of halide glass,^[^ ^20^ ^]^ mixed ions with different ionic radii and charges, such as alkaline earth cation Ca^2+^, Sr^2+^, Ba^2+^, can improve the disorder of network structure and glass‐forming ability. Accordingly, the introduction of a few covalent network‐forming polyhedra, e.g. adding [PO_4_] and [TeO_4_] into the AlF_3_‐BaCl_2_ system, can reduce the ionic characteristic of glass network and restrain the overlarge clustering of halide ions and anomaly grain growth.^[^ ^21^ ^]^ Note that the anomaly growth crystals can cause adverse light (Rayleigh) scattering loss and thus the reduction of glass transparency.^[^ ^22^ ^]^ Besides, the cracking resistance of ionic glass with a close‐packed structure is controlled by shear (plastic) flow‐mediated indentation deformation.^[^ ^23^ ^]^ The incorporation of small amounts of covalent oxide can increase different ionic species with varying ionic radii, such as [Al(F, Cl, O)_x_] and [Y(F, Cl, O)_x_] polyhedral, which can promote higher atomic packing, resulting in higher brittleness and cracking resistance.^[^ ^24^ ^]^ Accordingly, those will produce BaCl_2_: Eu nanocrystals containing glass‐ceramics with improved transparency and cracking resistance, then high resolutions and high light yields can be expected for their X‐ray imaging application, as shown in Scheme 1.

**Figure 1:**
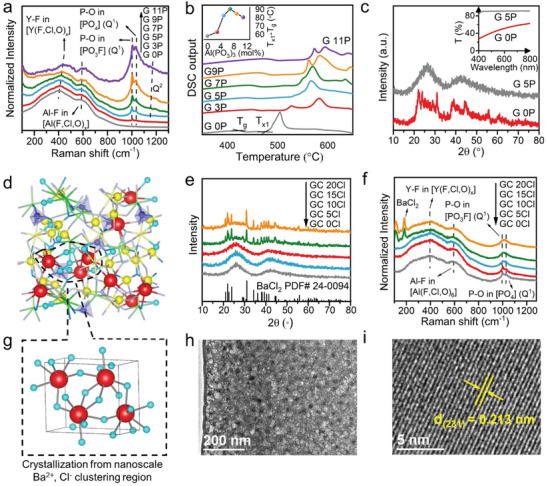
Theoretical design and experimental characterization of transparent GC containing BaCl_2_ nanocrystals. a) Raman spectra of glass containing with varied Al(PO_3_)_3_ content; b) DSC curves of glass containing with varied Al(PO_3_)_3_ content, the inset shows the dependence of the T_x1_‐T_g_ with Al(PO_3_)_3_ content; c) XRD patterns and transmittance spectrum (inset) of glass with and without 5 mol.% Al(PO_3_)_3_; d) A snapshot of Al (yellow), P (blue), Ba (red), and Cl (cycan) in the AIMD simulated structure of fluorochloroaluminate‐phosphate glass, and the residual ions are displayed only in bond form in the interest of clarity; e) XRD patterns of GC containing with different BaCl_2_ content; f) Raman spectra of GC containing with different BaCl_2_ content; g) Crystal structure of BaCl_2_ nanocrystals that are crystallized from nano‐scale Ba^2+^ and Cl^−^ clustering region; h) TEM micrograph image of BaCl_2_ GC; i) HRTEM micrograph image of one BaCl_2_ nanocrystal in glass.

To validate our research strategy, a series of fluorochloroaluminate‐phosphate (FCAP) glasses and glass ceramics with the molar composition of (44‐x)AlF_3_‐36(CaF_2_‐SrF_2_‐YF_3_)‐(20‐y)BaF_2_‐xAl(PO_3_)_3_‐yBaCl_2_ (x = 0, 3, 5, 7, 9, 11; y = 0, 5, 10, 15, 20; named as G xP and G yCl respectively; Figure S1, Supporting Information) was practiced to get uniform precipitation of BaCl_2_ nanocrystals in glass hosts. Fluorochloroaluminate (FCA) glass network comprises corner and edge‐linked [Al(F, Cl)_x_] and [Y(F, Cl)_x_] polyhedral with Ca^2+^, Sr^2+^, Ba^2+^ and additional Cl^−^ ions filling irregular space among these polyhedral. The introduced P^5+^ can bond with either O or F or Cl to form [P(O, F, Cl)_4_] tetrahedron, which can connect with [Al(F, Cl, O)_x_] and [Y(F, Cl, O)_x_] polyhedral chain.^[^ ^25^ ^]^ The ^27^Al magic angle spinning (MAS) nuclear magnetic resonance (NMR) spectra further reveal that the environment of Al^3+^ ions is six‐coordinated predominantly, with a small amount of five‐coordination (Figure S2a, Supporting Information).^[^ ^26^ ^]^ Raman spectra (**Figure** **1**a) and ^31^P MAS NMR spectra (Figure S2b, Supporting Information) further indicate that the Q^n^ phosphorous species (n represents the number of P‐O‐P bond) in G 5P is mainly Q^1^ species, and the Q^2^ species is increased with the content of Al(PO_3_)_3_.^[^ ^27^ ^]^ The introduced Q^1^ and Q^2^ species can reduce the ionic characteristic of glass network and improve the glass forming ability. Characteristic temperatures including glass transition temperature *T*_g_, the first onset crystallization temperature *T*_x1_ and melting temperature *T*_m_ were determined from differential scanning calorimetry (DSC) curves to obtain the *T*_g_/*T*_m_ and *T*_x1_‐*T*_g_ as the indicators of the glass forming ability^[^ ^28^ ^]^ (GFA) during the melt‐quenching process and the glass stability^[^ ^29^ ^]^ (GS) during the heating process respectively (Figure 1b; Table S1, Supporting Information). It is shown that the *T*_g_/*T*_m_ is significantly increased with the introduction of Al(PO_3_)_3_, indicating the improvement of GFA.^[^ ^30^ ^]^ However, an excessive introduction of Al(PO_3_)_3_ may cause the ionic network depolymerization (Figure 1a) and new phase separation (Figure 1b), and thus decrease the GS. In addition, the excessive content of Al(PO_3_)_3_ in the glass requires a higher glass melting temperature, leading to the volatilization of the BaCl_2_ component. Eventually, 5 mol.% Al(PO_3_)_3_ addition is selected to modify the ionic glass network. The AlF_3_‐BaCl_2_ based ionic glass (G 0P) is partially crystallized and shows a low transparency due to light scattering loss, as Figure 1c showed, while the AlF_3_‐BaCl_2_‐Al(PO_3_)_3_ based ionic‐covalent hybrid glass (G 5P) is amorphous and presents a high transparency. Note that transparent GC requires the controlled crystallization of transparent glass by subsequent heat treatment, rather than uncontrolled crystallization during the glass forming stage.

The Ba^2+^‐Cl^−^ clusters are aggregated as the precursor domains of the BaCl_2_ nanocrystallization in FCAP glass, through a successive Cl^−^/F^−^ substitution. The ^27^Al and ^31^P MAS NMR spectra of the G 0Cl and G 15Cl have changed little (Figure S2a,b, Supporting Information), which means that Cl^−^ ions are less likely to bond with Al^3+^ and P^5+^ ions.^[^ ^31^ ^]^ The glass structure's snapshot and the calculated cations’ coordination number of the G 15Cl sample from ab initio molecular dynamics (AIMD) simulations further proved that Cl^−^ ions are preferentially bonded with Ba^2+^, Y^3+^, and Sr^2+^ ions (Figure 1d; Figure S3, Supporting Information). But there is no stable Ba‐Y‐Sr‐Cl compound at the heat‐treatment temperature.^[^ ^31b^ ^]^ The highest bonding percentage of the Ba‐Cl bond as well as their longest bond distance (Figures S4–S6, Supporting Information) and lowest dissociation energy suggest that the Ba^2+^, Cl^−^ cluster would be crystallized preferentially, and the most of surrounding Y^3+^, Sr^2+^ would be returned to the remaining glass matrix during the crystallization process. Accordingly, one can probably extrapolate that the successive substitution of Cl^−^ for F^−^ can facilitate the tendency of Ba^2+^, Cl^−^ clustering behavior and the clustering scale, and thus the BaCl_2_ crystallization tendency during heat‐treatment process. Through a two‐step heat‐treatment procedure (460 °C for 4 h and 510 °C for 10 min) of the above‐mentioned G yCl samples, a series of corresponding GC yCl (*y* = 0, 5, 10, 15, 20) samples were obtained. This Ba^2+^, Cl^−^ clustering controlled crystallization behavior was well verified by the X‐ray diffraction (XRD), Raman, and ^35^Cl MAS NMR spectra results. There were no diffraction peaks in XRD curves from all precursor glasses and low Cl^−^ concentration (≤20 mol.%) doped GC, indicating their amorphous structure (Figure S7, Supporting Information). In contrast, typical crystalline diffraction peaks were found in high Cl^−^ concentration (>20 mol.%) doped GC, which are in good agreement with that of the orthorhombic BaCl_2_ (space group Pnam (62), PDF No. 24–0094) structure. It is worth noting that, unlike in fluorochlorozirconate glass, no concomitantly second crystalline phases were observed in our designed glasses.^[^ ^32^ ^]^ Moreover, new Raman peaks appear at the 100–200 cm^−1^ region for high Cl^−^ concentration doped GC, which can be well assigned to the precipitated BaCl_2_ crystals (Figure 1f; Figure S8, Supporting Information).^[^ ^33^ ^]^ The ^35^Cl MAS NMR spectrum of GC 15Cl is narrower than that of the G 15Cl (Figure S2d, Supporting Information), indicating that the Cl^−^ ions occupy narrower range of sites which are probably mainly bonded to Ba^2+^ ions due to the crystallization of BaCl_2_ nanocrystals.^[^ ^34^ ^]^ The ^31^P MAS NMR spectra of the G 15Cl and GC 15Cl show that the Q^2^ content increased after BaCl_2_ nanocrystallization (Figure S2b, Supporting Information), suggesting that the glass network was repolymerized and strengthened.^[^ ^27^ ^]^ As an extra benefit, an appropriate BaCl_2_ molar fraction (up to 15 mol.%) included in the FCAP glass can also improve the GS due to the mixed‐anion effect (Figure S9, Supporting Information).^[^ ^35^ ^]^ All the GCs with different BaCl_2_ content maintain high transparency (Figure S10, Supporting Information). Transmission electron microscopy (TEM) revealed that the BaCl_2_ nanocrystals of varying diameters mainly from 12 to 30 nm are homogeneously distributed in amorphous glass (Figure 1h; Figure S11, Supporting Information). High‐resolution TEM image of one BaCl_2_ nanocrystal in glass clearly displayed the lattice fringe of (231) plane with the d‐spacing of 0.213 nm, suggesting a high‐quality growth of BaCl_2_ nanocrystals in the designed amorphous glass (Figure 1i).

**Figure 2:**
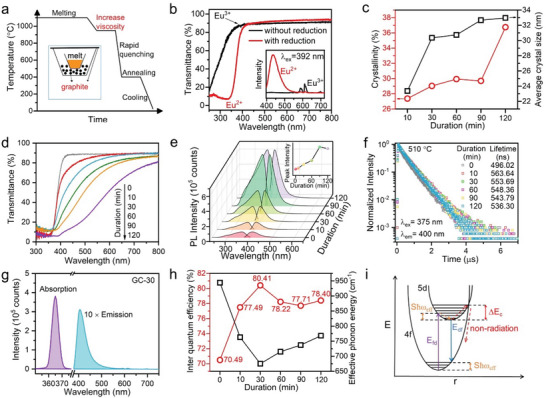
Spectroscopic behaviors of BaCl_2_: Eu^2+^ functionalized GCs. a) Transmittance spectra and emission spectra of glasses of the same batch composition fabricated with and without graphite reduction conditions; b) Transmittance spectra of BaCl_2_ GC with varied Eu doping concentration; c) Calculated crystallinity and average crystal size and d) transmittance spectra of BaCl_2_: Eu GCs with different heat‐treatment duration; e) Excited and emission spectra and f) Decay curves of BaCl_2_ GC doped with different heat‐treatment duration; g) Absorption and emission spectra of GC‐30 collected by an integrating sphere to evaluate quantum yields; h) Internal quantum efficiency and calculated average effective phonon energy of BaCl_2_: Eu GCs with different heat‐treatment duration; i) Configuration diagram of Eu^2+^ ions in BaCl_2_ nanocrystals.

The intense photoluminescence and short lifetime from the spin‐allowed 5d‐4f transition of Eu^2+^ is desired for PL and scintillator, while the forbidden 4f‐4f transition of Eu^3+^ leads to weak emission and long lifetime. For such purpose, we next control the valence state of divalent Eu ions, Eu doping concentration and crystallinity of BaCl_2_: Eu GC. Here we use a modified graphite powders‐based reduction method for reducing Eu^3+^ to Eu^2+^ in glass (**Figure** **2**a; Figure S12, Supporting Information). Note that an increase in the glass melt viscosity before the quenching process is essential for controlling the glass formation and improving the optical transmittance of glass. No characteristic absorption peak of Eu^3+^ ions at 392 nm is observed in BaCl_2_ GC, but it can be easily observed in the glass of the same composition without reducing condition (Figure 2b). Under excitation at 392 nm that is the strongest excited peak of Eu^3+^, the photoluminescence (PL) intensity of Eu^2+^ was much higher than that of Eu^3+^ (inset of Figure 2b), indicating the great reduction effect of the reduction method. The red‐shift of the cut‐off edge and the reduction of transmittance at 250–400 nm band in Eu doped BaCl_2_ GC further indicate the increase of Eu^2+^ concentration and the great reducing effect with varied Eu doping concentration in glass (Figure S13a, Supporting Information). With increasing Eu^2+^ ions concentration, the distance among Eu^2+^ ions becomes shorter and the non‐radiative energy transfer probability among Eu^2+^ ions would be increased, which consequently results in a typic concentration quenching phenomenon and the redshift of emission wavelength (Figure S13, Supporting Information).^[^ ^36^ ^]^ As the crystallization of BaCl_2_ nanocrystals is sensitive to the heat‐treatment duration in one‐step heat‐treatment process (Figures S14 and S15, Supporting Information), a two‐step heat‐treatment procedure (460 °C for 4 h and 510 °C for *x* min, named as GC‐*x*, *x* = 10, 30, 60, 90, 120) is carried out to get controllable precipitation of BaCl_2_: Eu nanocrystals in the glass. With the heat‐treatment duration prolonging, higher crystallinity of BaCl_2_: Eu nanocrystals with a larger average size can be precipitated from glass (Figure 2c), which are calculated by the well‐known Scherrer equation based on the XRD results (Figure S16, Supporting Information). From the standard results for Mie scattering in the Rayleigh limit,^[^ ^37^ ^]^ it can be derived that the optical extinction coefficient α for N independent spherical Rayleigh scattering particles per unit volume with refractive index n_1_ in a medium of refractive index *n*_m_ as

(1) $$\alpha \;=\frac{128}{9}\;NV\frac{\pi^{4}a^{4}}{\lambda^{4}}{\left(\Delta n\bar{n}\right)}^{2}$$

where *V* is the volume per particle (NV represents the crystallinity), *a* is the particle radius and the difference in refractive index Δ*n*  = |*n*_1_ − *n_m_* | is assumed to be much less than the average $\bar{n}=\;\left(n_{1}+n_{m}\right)/2$. Then, one can well know that the higher crystallinity and the larger crystal size of BaCl_2_: Eu^2+^ nanocrystals, the stronger Rayleigh scattering and the lower transparency of GC (Figure 2d).

With the heat‐treatment duration prolonging, the PL spectra of GCs exhibit a more similar characteristic with the BaCl_2_: Eu^2+^ single crystal, including short emission wavlength, small stokes shift and narrow full width at half maximum (FWHM) of emission bands (Figure 2e; Figure S17, Supporting Information). The integrated PL intensity is progressively enhanced with prolonged heat treatment duration but is weakened when the duration reaches 120 min, which may be caused by the above‐mentioned strong light scattering and low transparency of GC‐120 sample. The lifetime of Eu^2+^ ions in BaCl_2_ nanocrystal is larger than in glass due to lower phonon energy and is reduced with the heat‐treatment duration because of the enrichment of Eu^2+^ ions in BaCl_2_ nanocrystals (Figure 2e). The GC‐30 possesses the highest PL inter quantum efficiency (IQE) of 80.5% compared with 70.49% for corresponding precursor glass (Figure 2f,g; Table S2, Supporting Information). The improved and high IQE can be attributed to the low defect and ultra‐low phonon energy environment of BaCl_2_ nanocrystals which reduce the nonradiative recombination rates. The configuration coordinate model (Figure 2h), which has been successfully applied to various Eu^2+^ ions doped phosphors,^[^ ^38^ ^]^ can be used to clarify the relationship between phonon energy and PL. The nonradiative efficiency *Q*_nr_ can be described as the following equation:^[^ ^38b^ ^]^

(2) $$\;Q_{nr}=Q_{nr}^{0}\;\exp\left(\frac{-\Delta E_{c}}{kT}\right)$$

where is the pre‐exponential factor, *k* is the Boltzmann constant, *T* is the absolute temperature and ∆*E*_c_ is the thermal activation energy. In a simplified relationship, the ∆*E*_c_ is inversely proportional to the host phonon energy. Therefore, the Eu^2+^ ions enter into BaCl_2_ nanocrystal with lower phonon energy should have a lower non‐radiative rate and higher PL IQE. This relationship is further supported by the calculation of Huang–Rhys electron–phonon coupling factor *S* and the average energy of the local and effective phonon modes around Eu^2+^ ions ћω_eff_, which are derived from the Stokes shift, the FWHM of the emission bands (Table S3, Supporting Information) and Equation (2) in Ref. [39].

**Figure 3:**
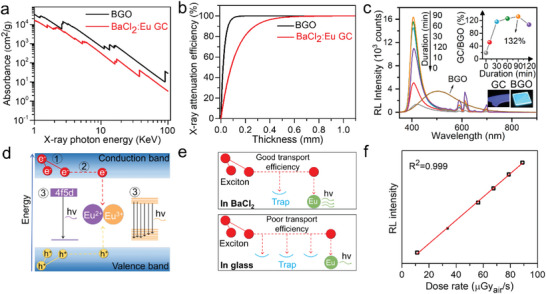
X‐ray scintillation properties and mechanisms of BaCl_2_: Eu functionalized GCs. a) Calculated X‐ray absorption coefficients of BaCl_2_: Eu GC and BGO as a function of X‐ray energy; b) X‐ray attenuation efficiency as a function of thickness. c) RL intensity of BaCl_2_: Eu GC with different heat‐treatment duration, corresponding precursor glass and commercial BGO crystal, the inset shows the steady state light yield calculation of BaCl_2_: Eu GC compared with BGO; d) Schematic illustration of the radioluminescence mechanism of the BaCl_2_: Eu GC; e) Difference between radioluminescence process in BaCl_2_ crystal and glass matrix. f) RL intensity of BaCl_2_: Eu GC dependence on X‐ray dose rate.

The BaCl_2_: Eu GC has been further evidenced as a bright scintillator with higher light yields than commercial Bi_4_Ge_3_O_14_ (BGO) single crystal and most of other glass and GCs (Table S4, Supporting Information). **Figure** **3**a shows the calculated absorption coefficients of BaCl_2_: Eu GC and BGO. The attenuation efficiency of BaCl_2_: Eu GC dependence on thickness compared with BGO was shown in Figure 3b, where the X‐ray is almost fully attenuated at the thickness of 0.6 mm. We then estimated the X‐ray light yield of BaCl_2_: Eu GCs scintillator with 1 mm thickness under steady state X‐ray illumination, using a 1 mm thickness commercial BGO as the reference standard that have the same X‐ray attenuation efficiency (Figure 3c; Figure S18, Supporting Information). In this case, the comparison of integrated RL intensity can represent the relative light yield in the steady‐state mode.^[^ ^40^ ^]^ The integrated RL intensity is significantly increased with the heat‐treatment duration prolonging to 90 min because of the increased BaCl_2_: Eu crystallinity, and is finally decreased due to the light scattering loss and low transparency of the GC‐120 sample. Although the glass matrix weakens the overall X‐ray absorption of the BaCl_2_: Eu GC which is weaker than the BGO single crystal, the X‐ray steady‐state light yield of the GC‐90 sample can reach about 10 824 photons MeV^−1^ by taking 132% times 8200 photons MeV^−1^ (BGO). This is because the superior BaCl_2_: Eu^2+^ nanocrystals have higher radioluminescence efficiency than the BGO crystal, which is supported by a higher light yield of BaCl_2_: Eu^2+^ single crystal than the BGO single crystal.^[^ ^12^ ^]^ The scintillation process can be divided into three basic processes: conversion, transport and luminescence (Figure 3d). The interaction of X‐ray with heavy atoms through the photoelectric effect, relaxation, and thermalization of the resulting electron and hole (e–h) pairs (excitons). The excitons can be transported to luminescent activators or quenching traps. The light yield can be estimated using the equation (LY = E/βE_g_*S*Q), where E is the deposited energy of X‐ray, βE_g_ is the average energy required to generate a single thermalized *e*–*h* pair, *S* is the transport efficiency of the *e*–*h* pair to the luminescent activators, and *Q* is the photoluminescence quantum efficiency of activators (IQE). First, the heavier Cl anion and lower phonon frequency of BaCl_2_ than that of fluoroaluminate‐phosphate glass host enable slow thermalization and consequently a higher order nonlinear quenching process in BaCl_2_, which has a lower nonradiative loss.^[^ ^41^ ^]^ Second, there are more traps and a much shorter mean free path of excitons in disordered glass matrix than in ordered BaCl_2_ nanocrystals,^[^ ^42^ ^]^ leading to a higher transport efficiency in BaCl_2_ nanocrystals (Figure 3e). Furthermore, the RL is much more sensitive to the valence state of Eu ions than PL (Figure 3c) due to the weak absorption effect of Eu^3+^ ions in PL (Figure 2b). Most of the UV excitation light is absorbed by Eu^2+^ ions and less by Eu^3+^ ions in PL, while the energy of dopant ions is transported from excitons in RL. Undoped BaCl_2_ GC only exhibits a weak and broadband spectrum (Figure S19a, Supporting Information) that may originate from self‐trapped excitons of BaCl_2_ nanocrystals.^[^ ^43^ ^]^ Therefore, the excellent steady‐state light yield can be attributed to the combined effect of high crystallinity of BaCl_2_: Eu nanocrystals, great reduction effect from Eu^3+^ to Eu^2+^ ions (Figure S19b, Supporting Information) and high PL IQE of Eu^2+^ ions in BaCl_2_ nanocrystals. The RL intensity of AlF_3_‐based BaCl_2_: Eu GC (GC‐30) has also proven to be much higher than that of traditional ZrF_4_‐based BaCl_2_: Eu GC (Figure S19d, Supporting Information). The integrated RL intensity of BaCl_2_: Eu GC was linearly correlated with the dose rate of X‐ray (Figure 3f), enabling a good energy resolution of X‐ray imaging application. Moreover, for collecting the reflection light other than transmitted light from GC, the RL intensity of GC‐120 could be as high as 313% of BGO single crystal (Figure S19e, Supporting Information), indicating its good potential application as X‐ray plate detectors.

**Figure 4:**
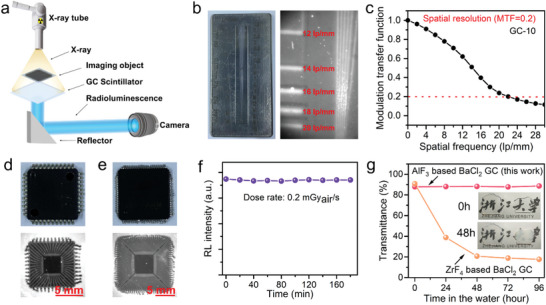
High‐resolution X‐ray imaging demonstration based on the BaCl_2_: Eu functionalized GC scintillator. a) Schematic showing the X‐ray imaging system. b) bright field and corresponding X‐ray images of a standard X‐ray test pattern plate using the GC‐10 as a scintillator. c) Modulation transfer function of the GC‐10 scintillator. d,e) bright field and corresponding X‐ray images of two chips with different sizes. f) Integrated RL intensity of Eu^2+^ activated BaCl_2_ GC under X‐ray irradiation for 180 min (dose rate: 0.2 mGy_air_ s^−1^, voltage: 50 kV). g) Change of transmittance at 500 nm of AlF_3_‐based BaCl_2_ GC and ZrF_4_‐based BaCl_2_ GC over time in the water. The inset shows the photograph of AlF_3_‐based BaCl_2_ GC (left) and ZrF4‐based BaCl_2_ GC (right) before and after soaking in the water for 48 h.

We then constructed a high‐resolution X‐ray imaging system by using the fabricated BaCl_2_: Eu scintillator (**Figure** **4**a). A sample for imaging was mounted between the X‐ray source and BaCl_2_: Eu GC scintillator. The X‐ray images of the standard test pattern plate by using GC‐10, GC‐30 and GC‐90 as screen have been shown in Figure 4b; Figure S20, Supporting Information. It is found that GC‐10 achieves the highest X‐ray imaging resolution of about 20 lp mm^−1^ since it contains less amount of luminescent nanocrystals but higher transparency. As heat‐treatment time increases indicating a higher degree of crystallization, GC‐30 and GC‐90 undergo radioluminescence increase but transparency decrease, consequently delivering X‐ray imaging resolution of 13 and 6 lp mm^−1^, respectively. The spatial resolution of GC‐10 is also determined to be about 22 lp mm^−1^ by modulation transfer function (MTF) that is in line with the result from the X‐ray test pattern plate. The X‐ray imaging resolution is mainly determined by the transparency of the GC scintillator, which may be because their radioluminescence is sufficiently high. We employed GC‐10, the most transparent BaCl_2_: Eu GC with heat‐treatment at 510 °C, to test several imaging objects, whose internal structure cannot be seen directly by naked eyes. X‐ray imaging enables clear inspection of the inner electronic components of a circuit board, the spring or screw inside a capsule, and the multiple silver holders inside chips with good contrast (Figure 4d,e;Figure S21, Supporting Information).

The designed AlF_3_‐based BaCl_2_: Eu GC scintillator exhibits outstanding X‐ray imaging performances with stable or long‐term operation potential (radioresistance, chemical and thermal stability) in the air and harsh environment. The RL intensity of GC‐10 was almost unchanged (Figure 4f) under a low dose rate of 0.2 mGy_air_ s^−1^ for 180 min of continuous X‐ray radiation. Interestingly, the RL intensity of GC‐10 was progressively increased under a high dose rate of 5 mGy_air_ s^−1^ for 60 min of continuous X‐ray radiation (Figure S22, Supporting Information). More specifically, this RL enhancement came from Eu^2+^ emission, whose energy may be transferred from the electron traps that are created by X‐ray irradiation and trapped in BaCl_2_ nanocrystals.^[^ ^44^ ^]^ Moreover, the thermal stability of GC‐10 in terms of PL intensity remains unaffected after heating treatment at 150 °C for 120 min (Figure S23, Supporting Information). Similarly, the RL intensity of GC‐10 was progressively and slightly increased with continuous heating at 100 °C for 60 min (Figure S24, Supporting Information). The AlF_3_‐based BaCl_2_: Eu GC‐10 scintillator remains high transparency after soaking in the water for several days, while the transmittance of a traditional ZrF_4_‐based BaCl_2_: Eu GC is readily reduced under water (Figure 4g; Figure S25, Supporting Information). The GC‐10 remains bright PL immersed in water at 85 °C for 10 h (Figure S26, Supporting Information), and the RL intensity of GC‐10 was almost unchanged (Figure S27, Supporting Information) after soaking in water for 144 h. The large improvement of water resistance in AlF_3_‐based glass can be mainly attributed to a water corrosion behavior on the glass surface induced by pH changes. As previously reported,^[^ ^45^ ^]^ the successive hydrolysis of ZrF_4_ can decrease the solution pH that greatly accelerates the glass dissolution, leading to a thick and peelable corrosion layer covered with precipitated ZrF_4_ and ZrBaF_6_ crystals. In comparison, the dissolution of AlF_3_‐based glass can slightly increase the solution pH, and the low F mobility in AlF_3_‐based glass prevents ion exchange and interdiffusion between F^−^ and OH^−^ ions are limited, causing a thin and stable corrosion layer without surface crystallization or microcracks. All the above stability testing indicates excellent feasibility of BaCl_2_: Eu GC scintillator for operating in the air and extreme environment.

## 3 Conclusion

In summary, we have demonstrated an ionic‐covalent hybrid network strategy to precipitate a high fraction of BaCl_2_: Eu^2+^ nanocrystals (NCs) in AlF_3_‐based glass‐ceramics (GC) as bright scintillators. The GCs containing BaCl_2_: Eu NCs exhibit a high fraction (the maximum crystallinity: ≈37%) of BaCl_2_: Eu nanocrystal scintillators with ultralow phonon energy (≈200 cm^−1^), high PL IQE (≈80.41%), and relatively small Stokes shift. Accordingly, this type of GC scintillators exhibits excellent scintillation performance, including highly efficient RL (132% compared with BGO crystal), high spatial resolution (6‐20 lp mm^−1^), and high stability against water, thermal, and irradiation. X‐ray imaging with the GC scintillators can unveil the inner structure of different objects with good contrast. It demonstrates its potential application in high‐quality X‐ray imaging. The results may trigger one new design approach to develop excellent metal halide crystal and glass composite scintillation materials.

## 4 Experimental Section

### Materials Preparation

The precursor glass was prepared by the traditional melting‐quenching method. A 15 g of reagent‐grade raw materials including AlF_3_, CaF_2_, SrF_2_, BaF_2_, BaCl_2_, YF_3_, Al(PO_3_)_3_ and EuF_3_ were well mixed, ground and put into a small alumina crucible. Then the small crucible was put into a big alumina crucible covered with a graphite lid, in which appropriate graphite powders were below the small crucible. After melting at 1100 °C for 25 min and 950 °C for 5 min, the precursor glass was made by pouring the melt onto a preheated brass plate and pressing it with another preheated brass plate. Note that the significance of reducing the temperature to 950 °C was to increase the viscosity of the glass melt and improve the glass forming and modeling ability. Subsequently, the glass was annealed in a muffle furnace at 420 °C for 2 h to reduce the internal stress of the glass and prevent cracking. The formation and size of BaCl_2_ nanocrystals in glass were controlled by one step or two‐step heat treatment process (Figure S14, Supporting Information), using a box‐type temperature‐controlled furnace. The nucleation temperature and crystallization temperature range were first determined between the characteristic *T*_g_ and *T*_x1_ according to differential scanning calorimetry results and were optimized by trial and error at intervals of 10 °C. A low heating rate was adopted during the heating‐crystallization process due to the highly‐sensitive crystallization behavior of BaCl_2_ nanocrystals.

### Structural, Thermal, and Morphological Properties Characterizations

Differential scanning calorimeter (DSC) curves were carried out on a SDT Q600, from 50 to 800 °C. Raman spectra were recorded on a Raman Laser confocal Raman spectrometer (LabRAM HR Evolution made by Horiba Jobin Yvon) with the excitation of 532 nm laser. X‐ray powder diffraction (XRD) measurement was performed on Shimadzu XRD‐7000 with Cu‐Kα radiation. FEI Quata 3D FEG dual‐beam Focused ion beam micro‐nano machining (FIB) was used to prepare the BaCl_2_ GC slice for microstructural analysis. Transmission electron microscopy (TEM), High‐resolution TEM (HRTEM), and Selected area electron diffraction (SAED) analysis were performed on an FEI Tencai G2 F20 S‐TWIN field emission transmission electron microscopy operating at an acceleration voltage of 200 KV. Solid‐state NMR experiments were carried out on a Bruker Avance III HD 500 M spectrometer (11.7 T). The resonance frequencies were 130.4, 202.6, 470.8, and 49.1 MHz for ^27^Al, ^31^P, ^19^F and ^35^Cl, respectively. All the spectra were recorded using a 4‐mm Bruker magic angle spinning (MAS) probe. ^27^Al MAS spectra were obtained at a spinning speed of 12 kHz. The pulse length is 0.73 µs (10° liquid angle) with a recycle delay of 0.5 s. ^31^P MAS spectra were obtained at a spinning speed of 12 kHz. The 90°pulse length was 2.9 µs with a recycle delay of 640 s. ^19^F MAS spectra were obtained at a spinning speed of 12 kHz. The 90°pulse length was 5.1 µs with a recycle delay of 16 s. ^35^Cl MAS spectra were acquired at a spinning speed of 12 kHz. Rotor‐ synchronized Hahn echoes with a selective 90° pulse of 5.15 µs were employed for signal acquisition at a recycle delay of 0.1s. The pulse length was 5.15 µs (45°liquid angle) with a recycle delay of 0.1 s. Chemical shifts were referenced to 1 m Al(NO_3_)_3_ solution ( = 0 ppm), ADP ( = 1.12 ppm), AlF_3_( = −172.5 ppm) and 1 m NaCl solution ( = 0 ppm).

### Optical and Luminescent Properties Characterizations

Transmittance and absorption spectra were collected from the Hitachi U‐4100 UV–vis–NIR spectrometer. Photoluminescence excitation/emission spectra and decay curves were recorded on an FLSP920 spectrometer (Edinburgh Instrument Ltd., Livingston, UK) with excitation by a xenon lamp and an external ns 375 ns laser diode respectively. The PL inter quantum efficiency (IQE) measurements were performed using a photoluminescence quantum yield spectrometer (Hamamatsu, Quantaurs‐QY Plus C13534‐12). Radioluminescence (RL) spectra were measured in an experimental system constructed by an X‐ray tube (Amptek Inc, Mini‐X, Ag target and tube voltage was 50 kV) and a fiber‐coupled fluorescence spectrometer (Ocean Optics QE PRO), which collect the transmittance light. The RL collecting reflection light of an opaque BaCl_2_: Eu GC and BGO crystal (Figure S19e, Supporting Information) was tested by zolix OmniFluo960‐X‐ray fluorescence spectrometer with X‐ray source (25 kV, 200 µA). The calculation methods of steady‐state light yield and MTF have been introduced in Ref. [40, 46].

### X‐Ray Imaging

The construction of the X‐ray imaging system is shown in Figure 4a. The X‐ray source used in the system was the above‐mentioned Mini‐X X‐ray tube. The X‐ray dose rate was adjusted by changing the tube current and the distance of the tube from the object. A leakage‐and‐low‐level X‐ray ion chamber dose meter (Radcal Corporation 10 × 5‐180) was used to calibrate the dose rates under different conditions. A reflector was used to deflect the light path to eliminate the negative effects caused by X‐rays radiating directly to the camera. X‐ray images were finally captured using a sCMOS camera (Photometrics, Prime 95B) with 1200 × 1200 pixels (pixel size:11 µm).

### Calculation of AIMD

The simulated structure of fluoroaluminate‐phosphate and fluorochloroaluminate‐phosphate glasses were carried out by using Born‐Oppenheimer molecular dynamics (BOMD) simulations through the QUICKSTEP algorithm^[^ ^47^ ^]^ of the CP2K package,^[^ ^48^ ^]^ employing the Gaussian plane waves approach. The initial configurations were constructed and randomized by applying Packmol^[^ ^49^ ^]^ in a cubic box with a density of 3.6 g cm^−3^, where the numbers of the simulated fluoroaluminate‐phosphate and fluorochloroaluminate‐phosphate glass structure was fixed 162 atoms. To avoid the possible atomic coordinate overlap at the initial state, each system first was optimized by the density functional theory (DFT) with PBE exchange‐correlation functionals^[^ ^50^ ^]^ and D3 (BJ‐damping) dispersion correction^[^ ^51^ ^]^ before performing BOMD simulations. Goedecker‐Teter‐Hutter (GTH) pseudopotentials and a double‐zeta valence polarised (DZVP) basis set^[^ ^52^ ^]^ that was optimized for molecular calculations (MOLOPT)^[^ ^53^ ^]^ were utilized, and the cutoff was set at 550 Ry. Periodic boundary conditions were used throughout the simulations.

The melt‐quench technique involves the initial heating up of the system at a rate of 3.0 K fs^−1^ to a high temperature of 3000 K and holding the melt at this temperature for 10 ps to relax the melt. The molten structure was subsequently cooled at a rate of 5.5 K fs^−1^ to 1400 K corresponding to experimental glass melting temperature and hold it for 12 ps, and then cooled at a rate of 2.0 K fs^−1^ to 800 K corresponding to glass transition temperature and hold it for 12 ps, and then cooled at a rate of 1.3 K fs^−1^ to 300 K corresponding to room temperature and hold it for 10 ps. The above processes were performed under the NVT (constant number of atoms, volume, and temperature) ensemble. To obtain a more reasonable structure, an NPT (constant number of atoms, pressure, and temperature) MD simulation of 12.5 ps was performed to maintain the temperature at 300 K and the pressure at 1.0 bar, which was consistent with the experimental conditions. Finally, all the structural properties were analyzed using the trajectories for production runs of the systems for 12.5 ps under NVT ensemble after geometrical and cell optimization.

Partial distribution functions g(r) and running coordinate number N(r) were calculated by a Rigorous Investigation of Networks Generated using Simulations (RINGS) code.^[^ ^54^ ^]^ The calculation formula of g(r) and N(r) can be found in Ref. [55].

## Conflict of Interest

The authors declare no conflict of interest.

## Supporting information

Supporting Information

## Data Availability

The data that support the findings of this study are available from the corresponding author upon reasonable request.
