# Supplementary material for: Bright Transparent Scintillators with High Fraction BaCl2: Eu2+ Nanocrystals Precipitation: An Ionic‐Covalent Hybrid Network Strategy toward Superior X‐Ray Imaging Glass‐Ceramics
Source: Adv Sci (Weinh). 2023 Oct 18;10(34):2304889. doi: 10.1002/advs.202304889 (PMC10700177; doi:10.1002/advs.202304889)
Supplement: Supplementary file 1 — Supporting Information [file ADVS-10-2304889-s001.pdf]

ADVANCED SCIENCE
Open Access

Supporting Information

for *Adv. Sci.*, DOI 10.1002/advs.202304889

Bright Transparent Scintillators with High Fraction $BaCl_2$: Eu^{2+} Nanocrystals Precipitation: An Ionic-Covalent Hybrid Network Strategy toward Superior X-Ray Imaging Glass-Ceramics

Qunhuo Liu, Peng Ran, Weilin Chen, Nian Shi, Wei Zhang, Xvsheng Qiao, Tingming Jiang*, Yang (Michael) Yang, Jinjun Ren, Zhiyu Wang, Guodong Qian and Xianping Fan*

Supporting Information

Bright transparent scintillators with high fraction $BaCl_2$: Eu^{2+} nanocrystals precipitation: an ionic-covalent hybrid network strategy towards superior X-ray imaging glass-ceramics

Qunhuo Liu, Peng Ran, Weilin Chen, Nian Shi, Wei Zhang, Xvsheng Qiao, Tingming Jiang*, Yang (Michael) Yang, Jinjun Ren, Zhiyu Wang, Guodong Qian and Xianping Fan*

Q. Liu., W. Zhang, X. Qiao, Z. Wang, G. Qian and X. Fan

State Key Laboratory of Silicon Materials,

School of Materials Science and Engineering,

Zhejiang University, Hangzhou, Zhejiang 310027, China

E-mail: qiaoxus@zju.edu.cn

P. Ran, T. Jiang and Y. (Michael) Yang

State Key Laboratory of Modern Optical Instrumentation,

College of Optical Science and Engineering,

Zhejiang University, Hangzhou, Zhejiang 310027, China

N. Shi and J. Ren

Key Laboratory of Materials for High Power Laser,

Shanghai Institute of Optics and Fine Mechanics,

Chinese Academy of Sciences, Shanghai, 201800, China

T. Jiang

School of Energy and Power Engineering

Chongqing University, Chongqing, 400044, China

E-mail: tingmingjiang@cqu.edu.cn

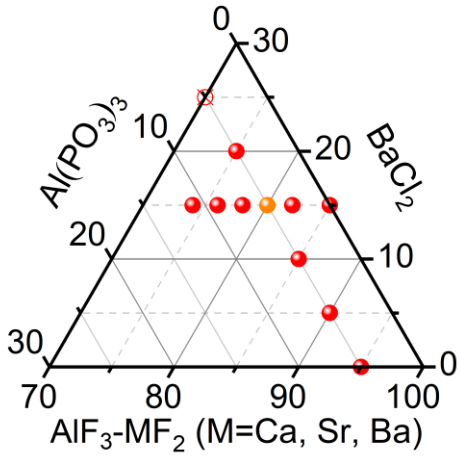

Figure S1 Glass composition design with different $BaCl_2$ and $Al(PO_3)_3$ content.

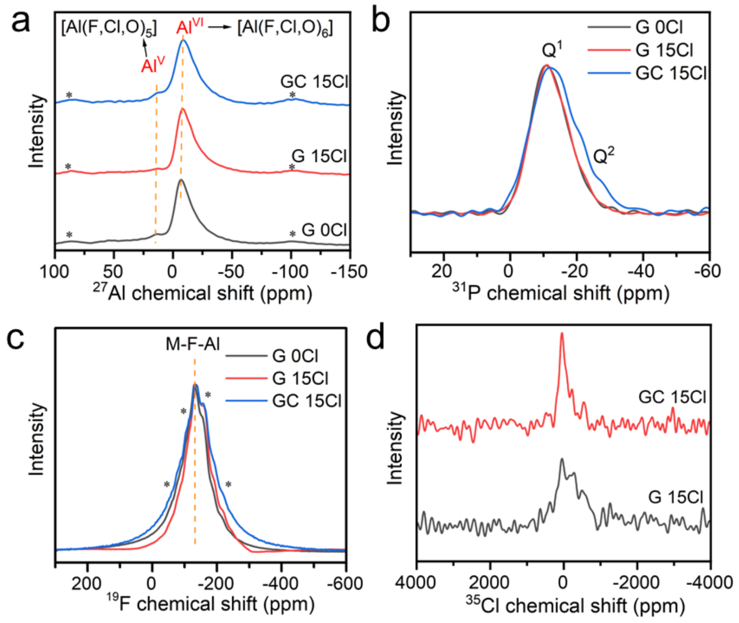

Figure S2 ^{27}Al, ^{31}P, ^{19}F and ^{35}Cl MAS NMR spectra of G 0Cl, G 15Cl and GC 15Cl samples. The asterisks in (a) and (c) represent rotating sidebands.

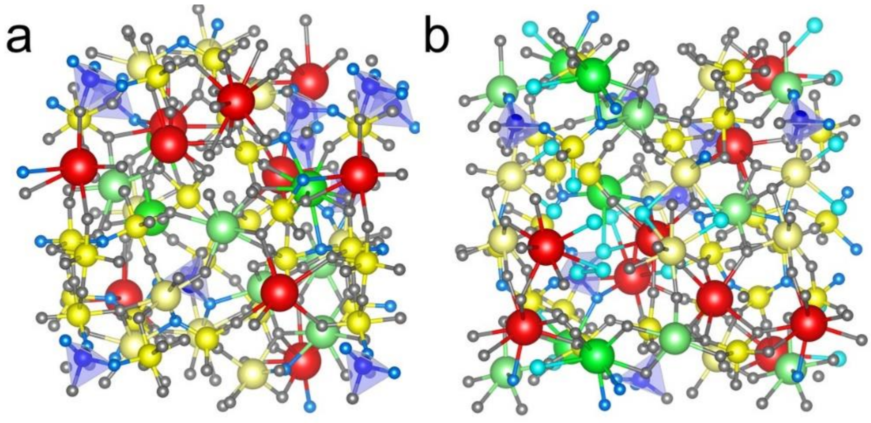

Figure S3 AIMD simulated structure of (a) G 0Cl and (b) G 15Cl samples.

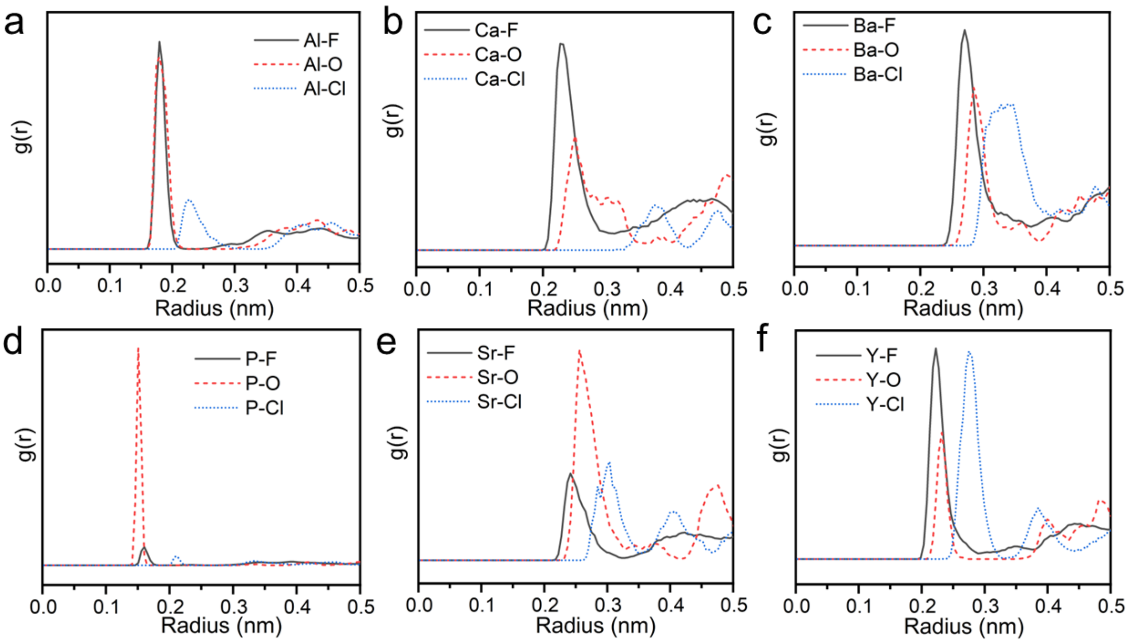

Figure S4 Pair distribution function g(r) for cation-anion in G 15Cl sample.

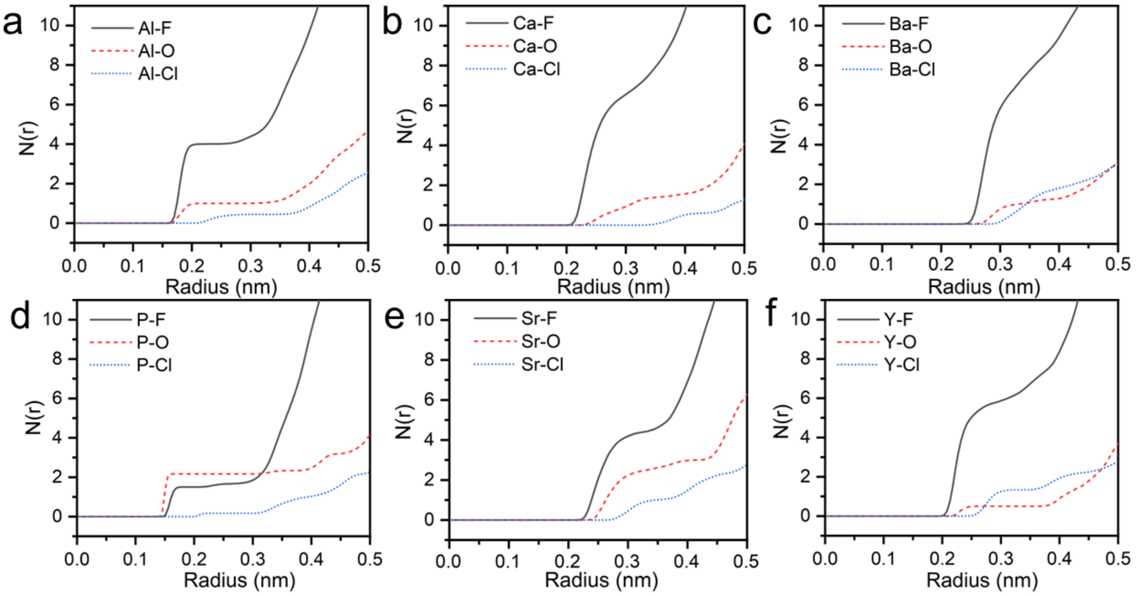

Figure S5 Running coordination number N(r) for cation-anion in G 15Cl sample.

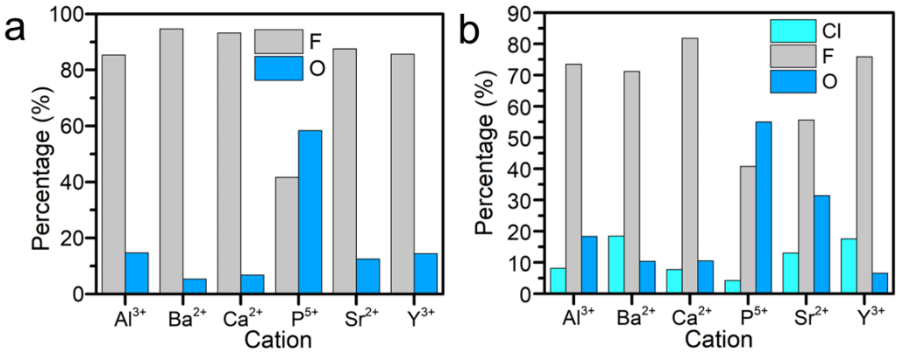

Figure S6 Statistic results of the coordination type for cation-anion in (a) G 0Cl and (b) G 15Cl sample.

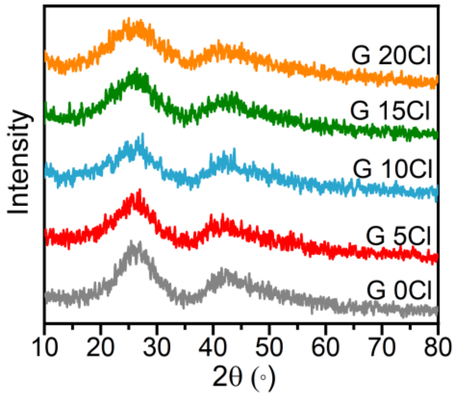

Figure S7 XRD patterns of precursor glasses and corresponding $BaCl_2$@glass containing with varied $BaCl_2$.

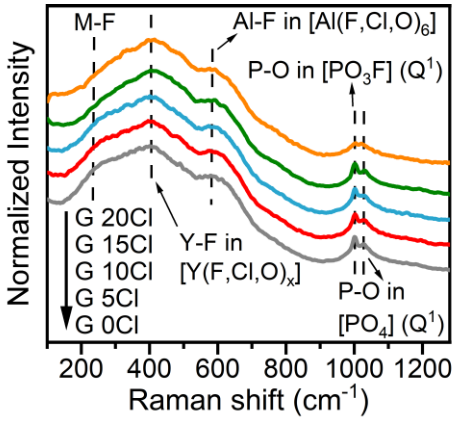

Figure S8 Raman spectra of glasses and glass ceamics with different $BaCl_2$ content

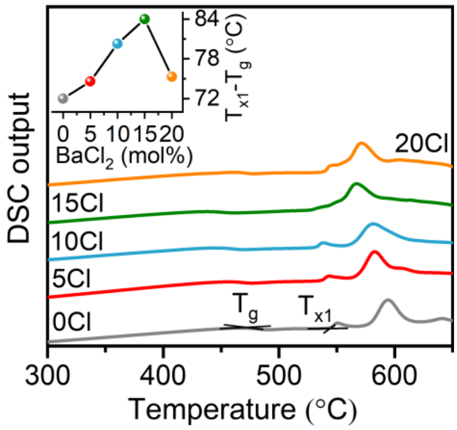

Figure S9 DSC curves of precursor glasses containing with different $BaCl_2$ content, the inset shows the varied characteristic temperature that reflect the glass forming ability and thermal stability.

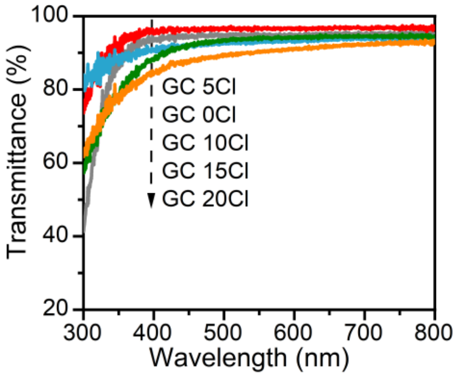

Figure S10 Transmittance spectra of GC with different $BaCl_2$ content.

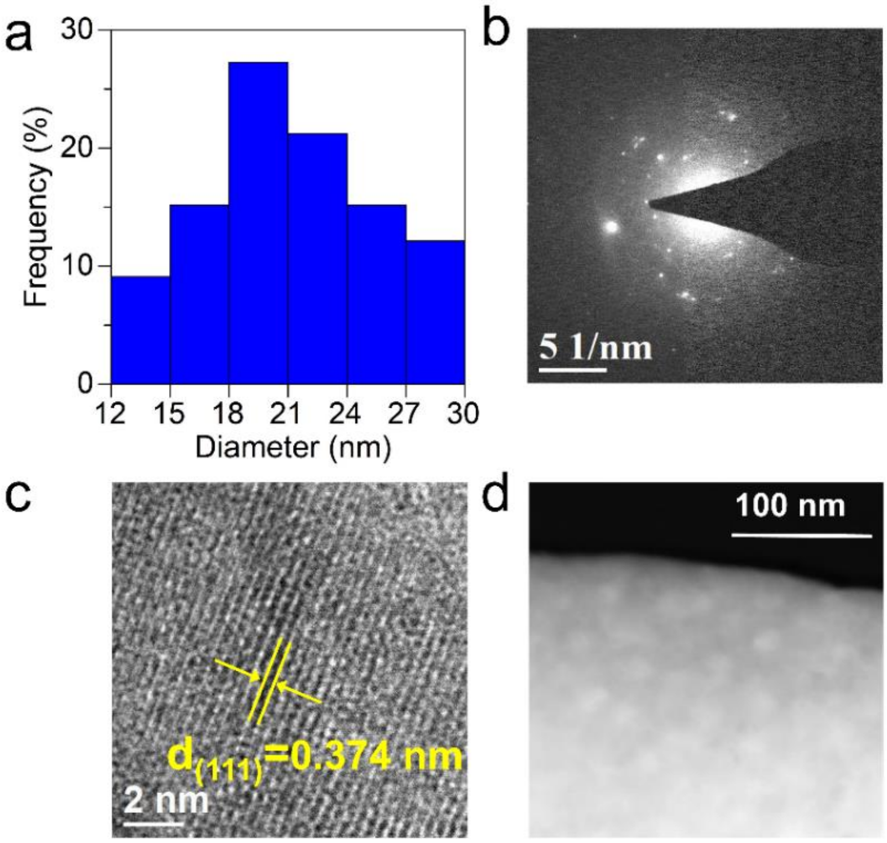

Figure S11 (a) size distribution of $BaCl_2$ nanocrystals dispersed in glass; (b) selected area electron diffraction (SAED) pattern, (c) HRTEM and (d) EDS mapping of the $BaCl_2$:Eu GC

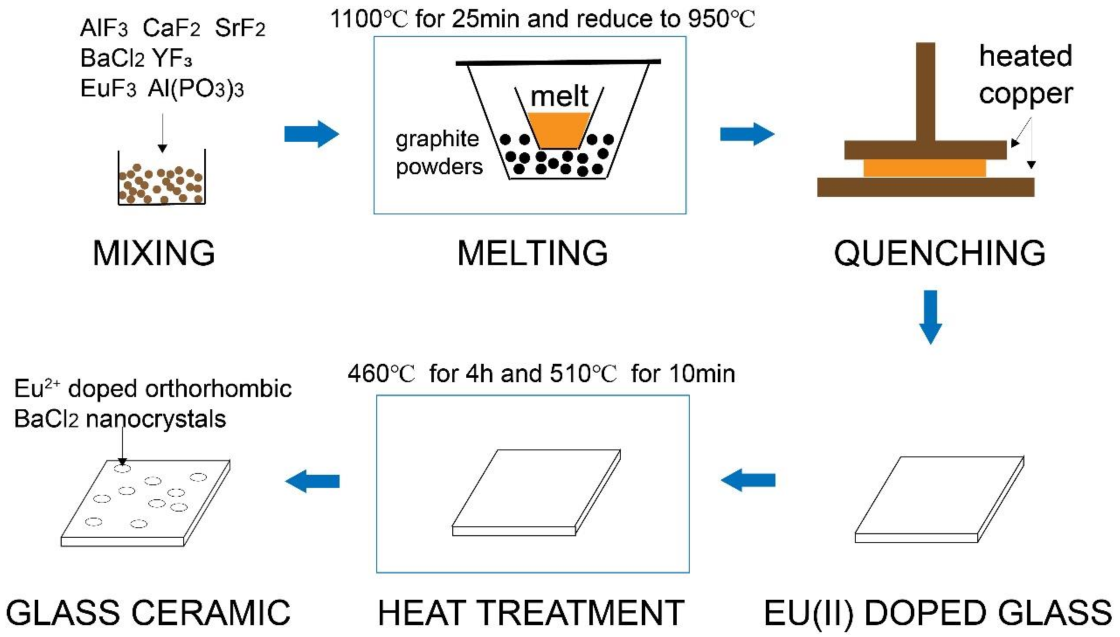

Figure S12 Preparation process of the $BaCl_2$: Eu using the modified graphite powders reducing method.

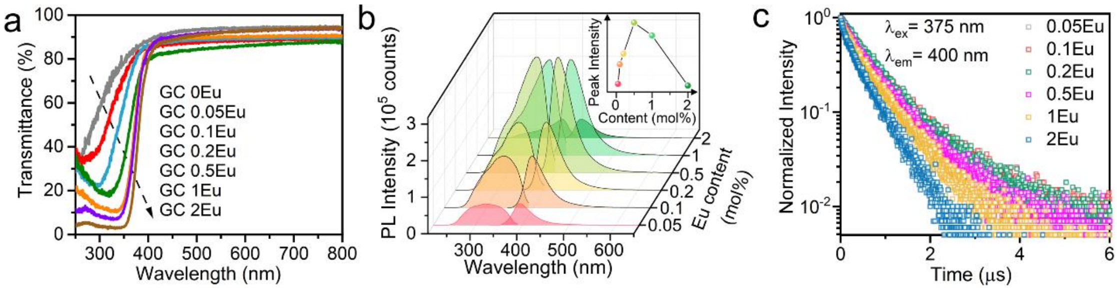

Figure S13 (a) PL spectra and (b) Eu^{2+} decay curves of 1Eu doped $BaCl_2$ GC with different heat-treatment duration (Two step heat-treatment: 460 °C 4h + 510 °C 10 min).

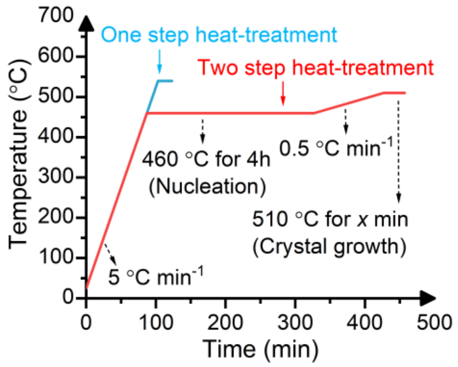

Figure S14 Typic one step heat-treatment condition and two-step heat-treatment condition for preparing $BaCl_2$: Eu GC.

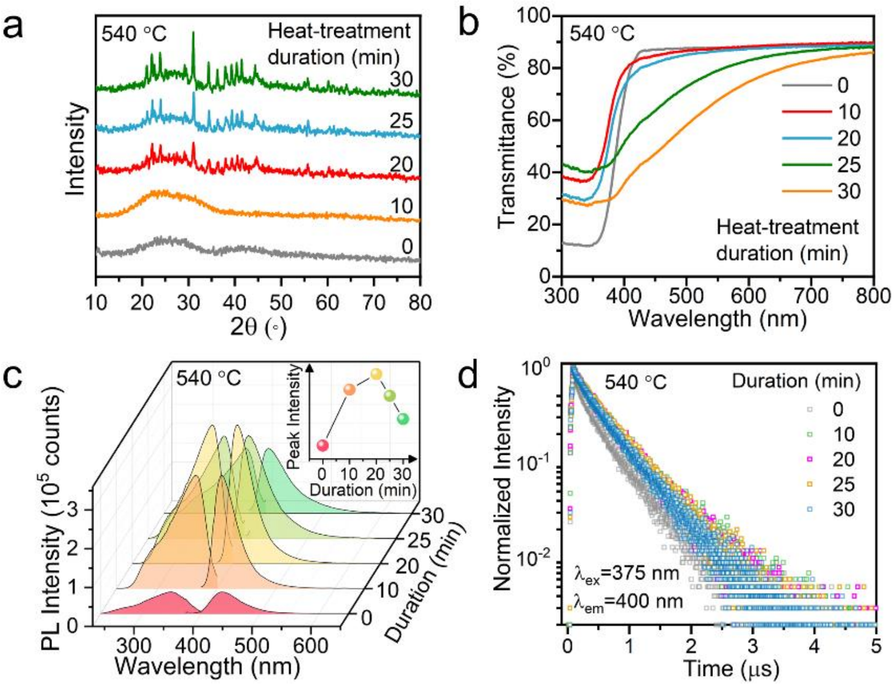

Figure S15 (a) XRD patterns, (b) transmittance spectra, (c) PL spectra and (d) Eu^{2+} decay curves of 1Eu doped $BaCl_2$ GC with different heat-treatment duration (One step heat-treatment).

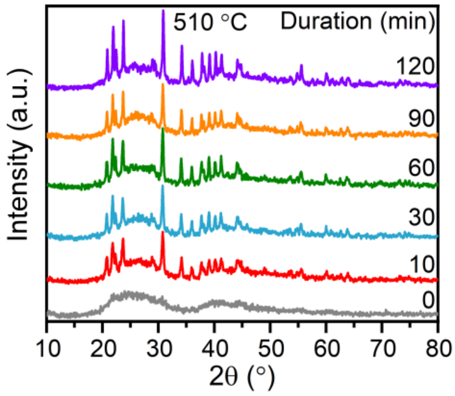

Figure S16 (a) XRD patterns and 1Eu doped $BaCl_2$ GC with different heat-treatment duration (Two step heat-treatment condition: 460 °C 4h + 510 °C x min).

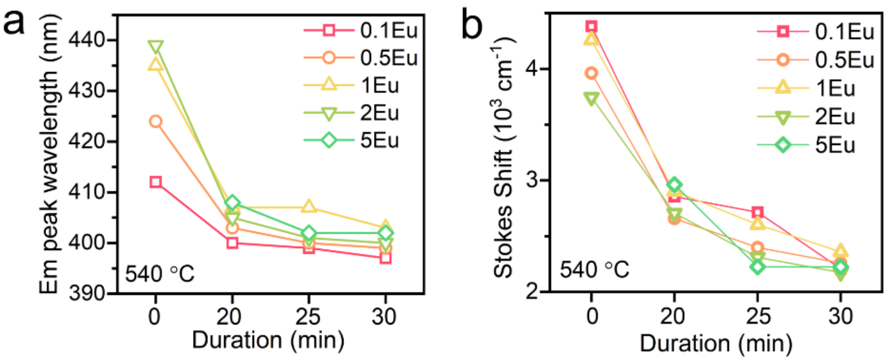

Figure S17 (a) Peak wavelength and (b) Stokes shift of PL of $BaCl_2$ GC with varied Eu doping concentration and different heat-treatment duration (One step heat-treatment).

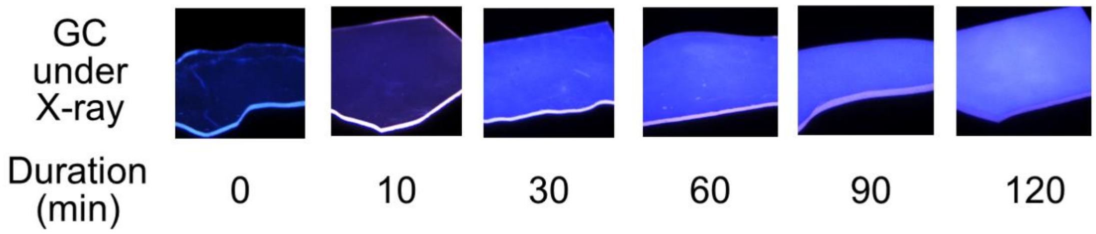

Figure S18 Photograph of $BaCl_2$: Eu GC with different heat-treatment duration under X-ray.

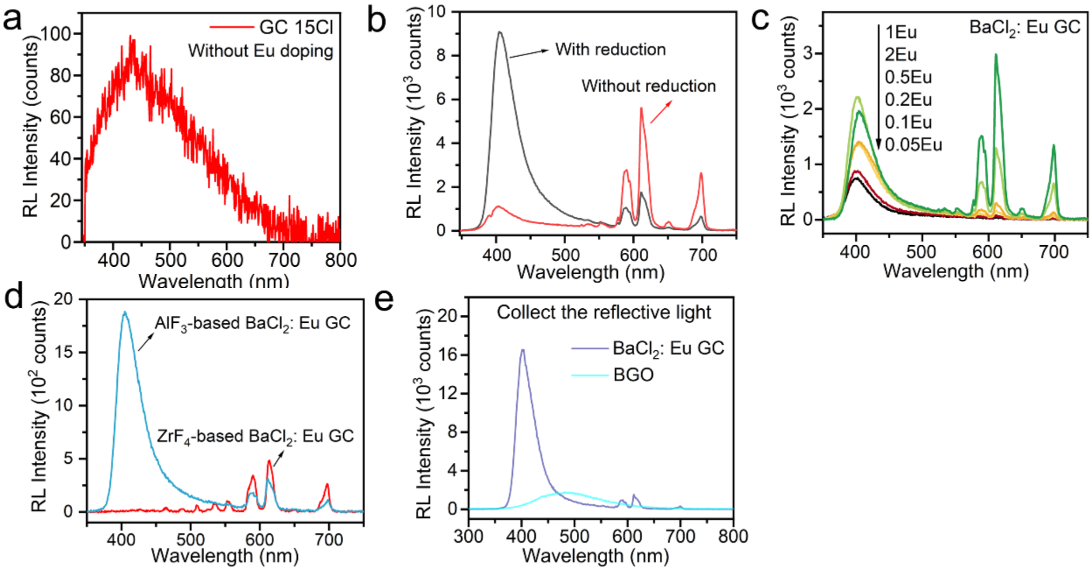

Figure S19 (a) RL intensity of $BaCl_2$ GC without Eu doping; (b) RL intensity of $BaCl_2$: Eu GC with and without graphite powders based reduction; (c) RL intensity of $BaCl_2$: Eu GC with different Eu doping concentration; (d) RL intensity of AlF_3-based $BaCl_2$: Eu GC and ZrF_4-based $BaCl_2$: Eu GC; (e) RL intensity of $BaCl_2$: Eu GC and commercial BGO by collecting the reflective light.

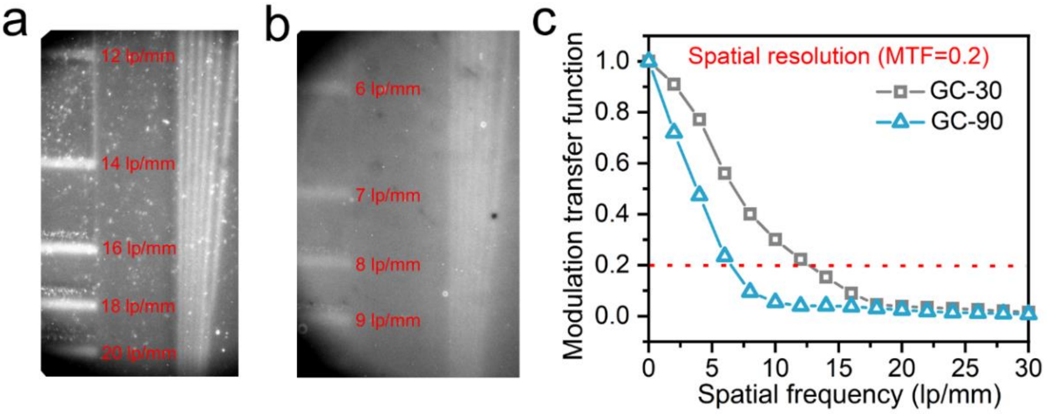

Figure S20 X-ray images of a standard X-ray test pattern plate using the (a) GC-30 and (b) GC-90 as scintillator. (c) Modulation transfer function of the GC-30 and GC-60 scintillator.

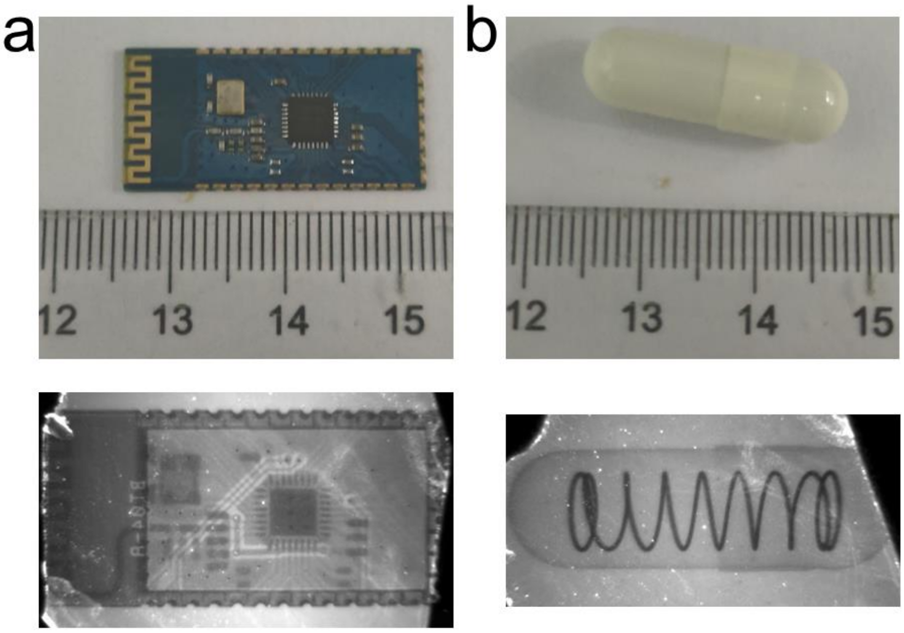

Figure S21 Bright field and corresponding X-ray images of (a) a circuit board and (b) a capsule containing with spring.

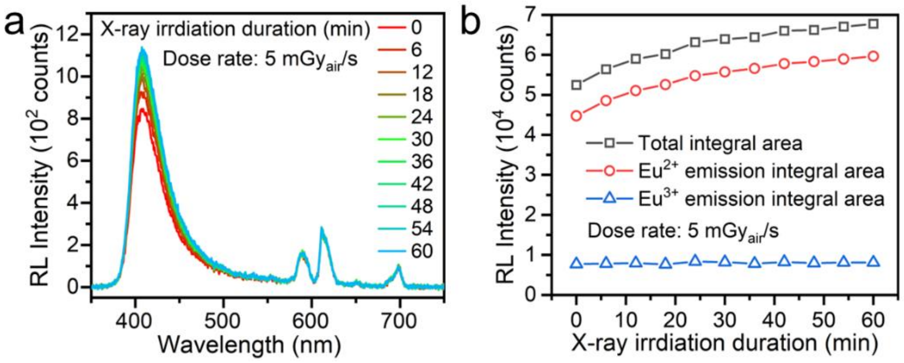

Figure S22 (a) In situ RL spectra and (b) integrated RL intensity of $BaCl_2$: Eu GC under X-ray irradiation for 60 min (dose rate: 5 mGy_{air} s^{-1}).

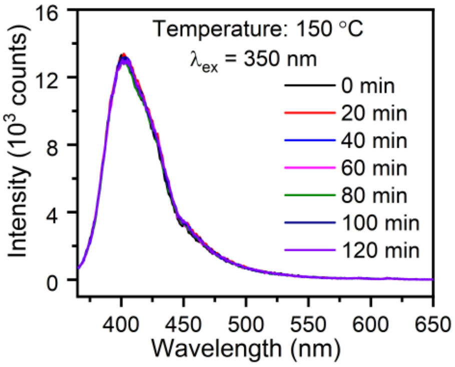

Figure S23 PL spectra of $BaCl_2$: Eu GC maintained at 150 °C for 120 min under UV light excitation.

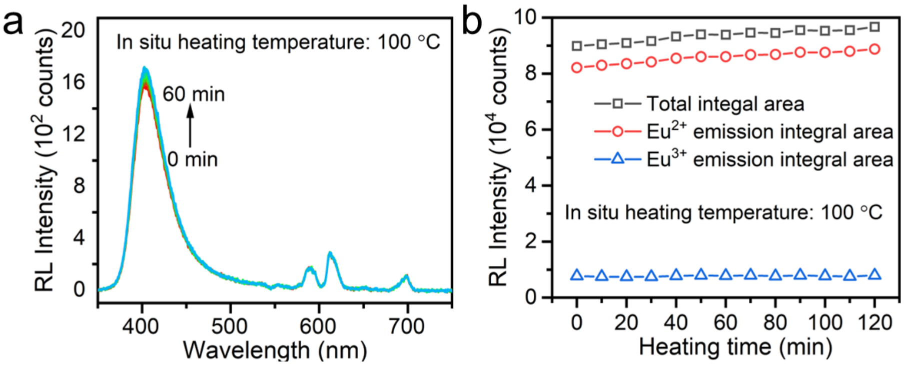

Figure S24 (a) In situ RL spectra and (b) integrated RL intensity of $BaCl_2$: Eu GC heating at 100 °C over time.

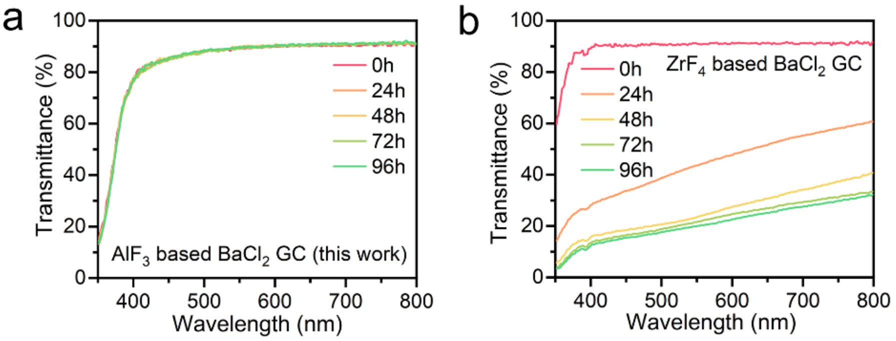

Figure S25 Transmittance spectra of (a) AlF_3 based and (b) ZrF_4 based $BaCl_2$ GC under water for 0, 24, 48, 72 and 96h.

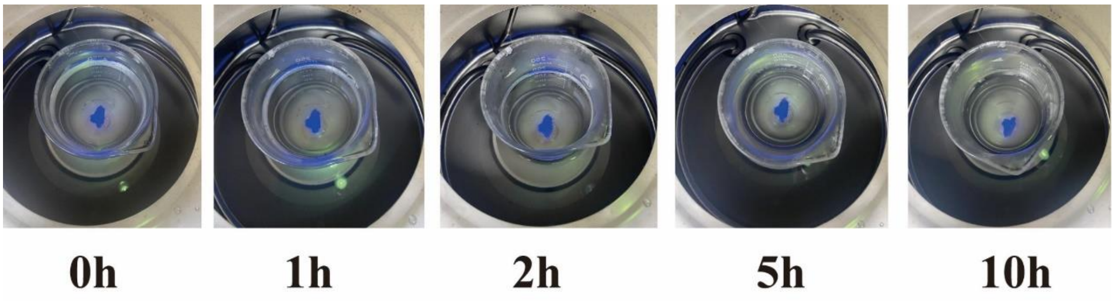

Figure S26 Photographs of $BaCl_2$: Eu GC in water maintained at 85 °C for 10 h under UV light excitation.

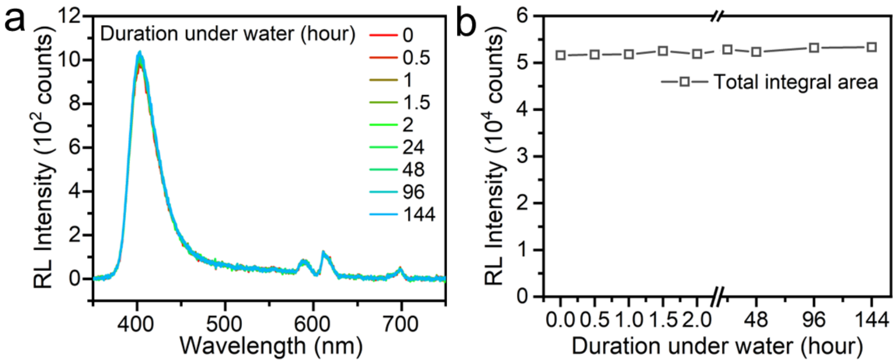

Figure S27 (a) In situ RL spectra and (b) integrated RL intensity of $BaCl_2$: Eu GC over time in the water.

Table S1 Characteristic temperature of glasses. The *glass was partially crystallization.

Glass	T_g (K)	T_{x1} (K)	T_m (K)	T_{x1}-T_g (K)	T_g/T_m
G 0Cl	744.6	816.7	1008.0	72.05	0.739
G 5Cl	730.4	809.6	1009.1	79.2	0.724
G 10Cl	724.8	805.1	1010.0	80.3	0.718
G 15Cl	713.5	800.4	1006.9	86.9	0.709
G 20Cl*	737.4	812.7	1000.5	75.3	0.737
G 0P	697.2	757.6	1013.8	60.4	0.688
G 3P	697.4	788.4	1000.3	91.0	0.697
G 5P	713.5	800.4	1006.9	86.9	0.709
G 7P	719.2	814.8	999.7	95.6	0.719
G 9P	740.3	826.8	1005.6	86.5	0.736
G 11P	750.9	833.1	1010.3	82.2	0.743

Table S2 Measured inter quantum efficiency of $BaCl_2$: Eu GC with different heat-treatment duration (Two step heat-treatment condition: 460 °C 4h + 510 °C *x* min).

Repeat	0 (glass)	10 min	30 min	60 min	90 min	120 min
1	70.566%	77.527%	80.453%	78.364%	77.745%	78.372%
2	70.529%	77.623%	80.410%	78.201%	77.828%	78.382%
3	70.491%	77.489%	80.410%	78.217%	77.711%	78.396%

Table S3 The spectroscopic parameters calculated from excitation and emission spectra.

Sample	0 (glass)	10 min	30 min	60 min	90 min	120 min
ΔS (cm^{-1})	3125	2543	2360	2236	2236	2100
FWHM (cm^{-1})	3293	2699	2457	2481	2521	2521
S	2.16	2.17	2.24	2.06	2.02	1.87
$\hbar\omega_{eff}$	944	763	675	715	737	768

Table S4 Scintillator of glasses and glass ceramics.

Glass host	Dopant	Nanocrystals	PL IQE	PL Lifetime	Integrated RL Intensity (BGO: 100%)	Ref.
Oxyhalide	/	$CsPbBr_3$	28.9%	15.2 ns	50%	[1]
Oxide	/	$Bi_4(GeO_4)_3$	/	/	33%	[2]
Oxyfluoride	Tb^{3+}	Sr_2GdF_7	59.1%	3.05 ms	194%	[3]
Oxide	Ce^{3+}	YAG	/	68.2 ns	43%	[4]
Oxyfluoride	Eu^{3+}	LaF_3	/	2.77 ms	20%	[5]
Oxide	Eu^{3+}	/	/	1.73 ms	13%	[6]
Oxide	Eu^{3+}	/	/	1.48 ms	4%	[7]
Oxide	Eu^{2+}	/	/	/	120%	[8]
Oxide	Eu^{2+}	$Ba_5Si_8O_{21}$	/	27.43 ns	246%	[9]
Fluoride-phosphate	Eu^{2+}	$BaCl_2$	80.41%	543.8 ns	313%	This work

References

[1] Y. Xu, X. Zhao, M. Xia and X. Zhang, *Journal of Materials Chemistry C*, **2021**, 9, 5452.
[2] S. Polosan and M. Secu, *Radiation Measurements*, **2010**, 45, 409.
[3] L. Teng, W. Zhang, W. Chen, J. Cao, X. Sun and H. Guo, *Ceramics International*, **2020**, 46, 10718.
[4]. M. Jia, J. Wen, W. Luo, Y. Dong, F. Pang, Z. Chen, G. Peng and T. Wang, *Journal of Luminescence*, **2020**, 221, 117063.
[5] J. Zhao, L. Huang, S. Zhao and S. Xu, *Optical Materials Express*, **2019**, 9.
[6] N. Wantana, E. Kaewnuam, B. Damdee, S. Kaewjaeng, S. Kothan, H. J. Kim and J. Kaewkhao, *Journal of Luminescence*, **2018**, 194, 75.
[7] X.-Y. Sun, X. Zhang, H.-H. Chen, Q.-L. Hu, W.-F. Wang, Z.-J. Zhang and J.-T. Zhao, *Journal of Non-Crystalline Solids*, **2014**, 404, 162.
[8] W. Chewpraditkul, Y. Shen, D. Chen, M. Nikl and A. Beitlerova, *Journal of Optoelectronics and Advanced Materials*, **2013**, 15, 94.
[9] J. Tang, S. Lv, Z. Lin, G. Du, M. Tang, X. Feng, J. Guo, X. Li, J. Chen, L. Wei, J. Qiu and S. Zhou, *Journal of Materials Science & Technology*, **2022**, 129, 173.
